# Detection of QTLs for genotype × environment interactions in tomato seeds and seedlings

**DOI:** 10.1111/pce.13788

**Published:** 2020-06-11

**Authors:** Nafiseh Geshnizjani, Basten L. Snoek, Leo A. J. Willems, Juriaan A. Rienstra, Harm Nijveen, Henk W. M. Hilhorst, Wilco Ligterink

**Affiliations:** ^1^ Wageningen Seed Lab, Laboratory of Plant Physiology Wageningen University Wageningen The Netherlands; ^2^ Theoretical Biology and Bioinformatics Utrecht University Utrecht The Netherlands; ^3^ Laboratory of Nematology Wageningen University Wageningen The Netherlands; ^4^ Bioinformatics Group Wageningen University Wageningen The Netherlands

**Keywords:** high phosphate, low nitrogen, maternal environment, QTL × E, seed quality, seedling establishment, tomato

## Abstract

Seed quality and seedling establishment are the most important factors affecting successful crop development. They depend on the genetic background and are acquired during seed maturation and therefor, affected by the maternal environment under which the seeds develop. There is little knowledge about the genetic and environmental factors that affect seed quality and seedling establishment. The aim of this study is to identify the loci and possible molecular mechanisms involved in acquisition of seed quality and how these are controlled by adverse maternal conditions. For this, we used a tomato recombinant inbred line (RIL) population consisting of 100 lines which were grown under two different nutritional environmental conditions, high phosphate and low nitrate. Most of the seed germination traits such as maximum germination percentage (G_max_), germination rate (t_50_) and uniformity (U_8416_) showed ample variation between genotypes and under different germination conditions. This phenotypic variation leads to identification of quantitative trait loci (QTLs) which were dependent on genetic factors, but also on the interaction with the maternal environment (QTL × E). Further studies of these QTLs may ultimately help to predict the effect of different maternal environmental conditions on seed quality and seedling establishment which will be very useful to improve the production of high‐performance seeds.

## Abstract 2

The environment in which seeds develop has profound effect on seed and seedling quality. To elucidate the underlying mechanisms of this effect in tomato, we performed QTL analysis with a RIL population grown in different nutritional maternal environments and detected many QTLs which are dependent on both genetic factors and the maternal environment.

## 1 INTRODUCTION

Tomato is one of the most important agricultural commodities due to the level of production throughout the world (4.8 million hectares with the average yield of 37 ton per hectare [FAOSTAT2016]) (Heuvelink, 2018). Moreover, tomato is of scientific importance as a model organism for fruit‐bearing plants (Giovannoni, 2001; Schauer et al., 2006). Tomato producers are attempting to produce plants with high quality fruits as well as with high resistance against stressful environments, such as high temperature (HT) and osmotic stress. Since tomato is propagated by seed, the first step to improve tomato production is improving the quality of the seeds.

One of the characteristics of seed quality is the ability of the seed to germinate quickly and uniformly, not only under optimal but especially also under stress‐full germination conditions (Foolad, Subbiah, & Zhang, 2008). Furthermore, seed quality is not solely determined by germination but also by many other attributes such as genetic purity, vigour, viability and lack of any disease and damages, which all affect seed performance (Hilhorst, Finch‐Savage, Buitink, Bolingue, & Leubner‐Metzger, 2010; Hilhorst & Koornneef, 2007; Hilhorst & Toorop, 1997). Additionally, these quality parameters may severely affect seedling establishment and further growth of the plant and, ultimately, the success of crop production. In general, low quality seeds, for instance seeds with low vigour, lead to poor seedling establishment and finally lower and non‐profitable crop yield (Finch‐Savage, 1995). An important determinant of seed quality and performance is the maternal environment (ME) under which seeds develop and mature. The different environmental factors during seed development, such as temperature, light quality and quantity as well as nutrients may affect ultimate seed quality. Therefore, seed quality is defined by both the genetics (G) and the environment (E), as well as their interaction (G × E) (Koornneef, Bentsink, & Hilhorst, 2002; McDonald, 1998).

In tomato, as in many other crops, the domestication process has been accompanied by an attrition of genetic variation and, consequently, loss of many potentially desirable traits (Doebley, Gaut, & Smith, 2006; McCouch, 2004). Therefore, domesticated cultivars are sensitive to non‐optimal germination conditions which limit their production to optimal environments (Foolad et al., 2008; Foolad & Lin, 1997, 1998). However, a large source of genetic variation is found within wild species of tomato, such as *Solanum habrochaitis*, *Solanum pimpinellifolium* and *Solanum pennellii*. As cultivated crops suffer from abiotic stress, such as HT, drought and salinity by increased frequency and severity due to climate change, existing genetic variation could be used to reintroduce lost valuable traits in the domesticated cultivars to cope with these environmental stresses (Kazmi et al., 2012; Lippman, Semel, & Zamir, 2007).

Seed dormancy is profoundly affected by the environment (Huo & Bradford, 2015). Seeds perceive their environment and under undesirable conditions they typically do not germinate and become dormant. Nowadays, due to global warming, HT is regarded as one of the most important unfavourable environmental factors affecting seed germination. For instance, the germination of seeds of several species such as carrot (*Daucus carota*), lettuce (*Lactuca sativa*) and Arabidopsis are affected by thermo‐inhibition or thermo‐dormancy (Geshnizjani, Ghaderi‐Far, Willems, Hilhorst, & Ligterink, 2018; Lafta & Mou, 2013; Nascimento, Huber, & Cantliffe, 2013; Toh et al., 2008). Thermo‐inhibition refers to the fact that seeds will stop germination under HT, yet will immediately germinate upon facing the optimal temperatures. In the case of seed dormancy, seeds will germinate neither at HTs, nor at subsequent lower/optimal germination temperatures (Argyris, Dahal, Hayashi, Still, & Bradford, 2008; Huo, Dahal, Kunusoth, McCallum, & Bradford, 2013). It is previously reported that different MEs such as temperature, light, water and nutrient availability during seed development and maturation may affect seed dormancy (Bewley, Bradford, & Hilhorst, 2012; Fenner, 1991; Hilhorst, 1995; Holdsworth, Bentsink, & Soppe, 2008).

Natural variation present in traits such as seed size and weight, as well as dormancy and germination, exhibits a continuous distribution and is considered as quantitative variation likely regulated by multiple quantitative trait loci (QTL) (Argyris et al., 2008; Koornneef et al., 2002). A population of recombinant inbred lines (RILs) may be used for measuring the existing natural variation followed by QTL mapping as a powerful tool to detect loci affecting seed traits (Alonso‐Blanco et al., 2009). Many studies have characterized QTLs regulating complex quantitative seed traits in different species, such as Arabidopsis, Tomato and Wheat (Argyris et al., 2008; Joosen et al., 2012; Kazmi et al., 2012; Koornneef et al., 2002; Mathews et al., 2008). However, few studies have been conducted to investigate the interaction between the ME and genetic variation (Dechaine, Gardner, & Weinig, 2009; Elwell, Gronwall, Miller, Spalding, & Durham Brooks, 2011; Geshnizjani et al., 2019; He et al., 2014; Postma & Agren, 2015). In general, final seed performance is determined by the function of several genes and their interaction with the environment. Using high throughput genetic tools, including QTL mapping, to discover the genotype by environment interaction effects on QTLs affecting these seed traits provides a better understanding of how plants adapt to and cope with new stressful environments and is a prerequisite for crop improvement (Des Marais, Hernandez, & Juenger, 2013; El‐Soda, Malosetti, Zwaan, Koornneef, & Aarts, 2014).

In this study we analysed natural variation of several seed and seedling traits including maximum germination percentage and rate of germination under control and stress conditions, as well as fresh and dry weight of seedlings and compare the results with the previously published thermo‐dormancy and ‐inhibition of seed germination characteristics (Geshnizjani et al., 2018). We have used a RIL population derived from two tomato accessions: *Solanum lycopersicum* (cv. Moneymaker) (MM) and *Solanum pimpinellifolium* (PI) (Voorrips, Verkerke, Finkers, Jongerius, & Kanne, 2000). From the collection of tomato wild cultivars, *S. pimpinellifolium* has been used most frequently in breeding programs as it is the most closely related wild species to the domesticated tomato cultivar (*S. lycopersicum*) and has also the ability to naturally cross with *S. lycopersicum*. To investigate the existing genetic variation of seed and seedling related traits, we specifically focused on the ME in which seeds develop and mature. We compared the identified QTL for seed and seedling traits between the different nutritional environments of the mother plant. To do so, the RILs were exposed to high phosphate (HP) and low nitrate (LN) environments during seed development and their seeds were tested for seed and seedling related traits. In addition we performed a QTL × E approach to increase the power for detecting the loci affected by the different MEs (Joosen et al., 2012; Malosetti, Voltas, Romagosa, Ullrich, & Van Eeuwijk, 2004; Moreau, Charcosset, & Gallais, 2004; Van Eeuwijk, Malosetti, & Boer, 2007). In this study we show, that the interaction between ME, gemination environment and specific genetic loci can affect seedling establishment.

## 2 MATERIALS AND METHODS

### 2.1 Plant material and growth conditions

The RIL population was derived from a cross between two parental lines: *S. lycopersicum* cv. Moneymaker and *S. pimpinellifolium* (accession CGN14498). The population of 100 lines was genotyped in the *F*_*7*_ using a set of 865 single nucleotide polymorphism (SNP) markers, described in Voorrips et al. (2000). *F*_*8*_ seeds of this population were grown under controlled conditions in a greenhouse at Wageningen University, the Netherlands with 16 hr light and 8 hr dark. The temperature was adjusted to 25°C during the day and 15°C during the night. All the lines were fertilized uniformly by the same dosage of nutrient until flowering (Tables S1–S10). From the first open flower onwards the lines were transferred to new nutritional conditions and exposed to high and low concentrations of phosphate and nitrate, respectively (HP: 14.0 mM nitrate, 10.0 mM phosphate; LN: 2.4 mM nitrate, 1.0 mM phosphate; Standard: 14.0 mM nitrate, 1.0 mM phosphate used in Kazmi et al., 2012, Table S1).

Afterwards, healthy full ripened fruits were collected and seeds were extracted. To remove the main part of the pulp that is stuck onto the seeds 1% hydrochloric acid (HCl) was used. The seed extract together with diluted HCl was passed through a mesh sieve and then washed with water to remove the residual pulp and HCl. In order to disinfect the seeds, they were soaked in a trisodium phosphate (Na_3_PO_4_·12H_2_O) solution and then dried on filter paper at room temperature for 3 days and brushed to remove impurities. At the end, the seeds were stored in small paper bags in a cold (13°C) and dry (30% RH) storage room (Kazmi et al., 2012).

### 2.2 Phenotyping of seeds and seedlings

#### 2.2.1 Seed size and weight

Seed size was measured by using a Nikon D80 camera fixed to a repro stand with 60 mm objective and connected to a PC with Nikon camera control pro software version 2.0 (Joosen et al., 2010). The images of 12‐hr imbibed seeds on white filter paper (20.2 × 14.3 cm) were processed by ImageJ (http://rsbweb.nih.gov/ij/) combining colour threshold with particle analysis. For seed weight, a batch of dry seeds was weighed and then divided by the number of the weighed seeds.

#### 2.2.2 Germination experiments

Germination experiments were executed in a randomized design with two replications of around 50 seeds per RIL, as well as the parental lines. The seeds were sown in germination trays (21 × 15 cm DBP Plastics, http://www.dbp.be) containing one layer of white filter paper (20.2 × 14.3 cm white blotter paper; Allpaper BV, Zevenaar, The Netherlands, http://www.allpaper.nl) and 15 mL of demineralized water for normal and HT conditions, or 15 ml NaCl (−0.5 MPa; Sigma‐Aldrich) or mannitol (−0.5 MPa; Sigma‐Aldrich) for salt and osmotic stress, respectively. Each germination tray was loaded with three samples using a special mask to ensure correct placement of the seeds. The trays were piled up with one empty tray containing one white filter paper and 15 ml of water at the bottom and top of the pile and a white plastic lid at the top. The trays were wrapped in a transparent plastic bag and stored at 4°C for 3 days and subsequently transferred into a dark incubator (type 5,042; seed processing Holland, http://www.seedprocessing.nl) at 25°C except for the HT condition, which was at 35°C. Germination was scored manually by counting the germinated seeds at 24‐hr intervals during 14 following days for salt and osmotic stress and at 8‐hr intervals for 1 week in the case of normal and HT conditions. In order to quantify seed vigour, we germinated tomato seeds in water and under three suboptimal conditions; NaCl and mannitol solutions, and HT.

#### 2.2.3 Seedling phenotyping

Seedling characteristics were measured by sowing around 20 seeds of each seed batch on germination trays containing two blue germination papers (5.6′ × 8′ Blue Blotter Paper; Anchor Paper Company, http://www.seedpaper.com) and 50 ml demineralized water. The germination trays were stored at 4°C for 3 days. Then, they were transferred to an incubator at 25°C without light. The first 10 germinated seeds were placed on circular blue filter papers (9 cm Blue Blotter Paper; Anchor Paper Company, http://www.seedpaper.com) which were placed on a Copenhagen table at 25°C in a randomized complete block design with two biological and two technical replicates, for 10 days. Conical plastic covers with a small hole on top were placed on top of each filter paper to inhibit evaporation. At the end of the 10 days, the seedlings were collected and fresh weight of their shoots and roots was measured (FWSH and FWR respectively). The dry weight of shoots and roots was also measured after incubating them at 80°C for 3 days (DWSH and DWR respectively). Average trait values per RIL per phenotype can be found in Table S2.

### 2.3 Statistical analysis

#### 2.3.1 Calculation of seed quality traits

Seed quality traits, G_max_ (maximum germination), t_10_^−1^ (reciprocal of time to reach 10% of maximum germination), t_50_^−1^ (germination rate, reciprocal of time to reach 50% of maximum germination), U_8416_^−1^ (uniformity, reciprocal of time between 16 and 84% of maximum germination) and area under the germination curve (AUC till 200 hr) were measured based on the cumulative germination data using the curve‐fitter module of the Germinator package (Joosen et al., 2010). The parameters t_10_^−1^, t_50_^−1^ and U_8416_^−1^ were only determined when germination of more than 80% of the RILs reached 10, 50 and 84%, respectively. The average of two biological replicates of each line was used for subsequent QTL analysis.

#### 2.3.2 Broad sense heritability, coefficient of variation and ANOVA analysis

The total phenotypic variation (V_P_) can be affected by genetic (V_G_) and environmental (V_E_) variation (V_P_ = V_G_ + V_E_). For each maternal and germination environment (GE) the broad sense heritability (H^2^) was calculated for individual traits as the proportion of phenotypic variation due to the effect of genetic variation (H^2^ = V_G_/V_P_). The calculation was performed in Genstat 18 with the QTL phenotypic analysis tools, using preliminary single environment analysis and considering plant replications as an additional fixed term. Within a population the absolute variation or dispersion per trait is defined as the standard deviation (σ). The relative variation called the coefficient variation (CV) for individual traits is the ratio of the standard variation to the mean (μ) of the lines in the population (CV = [σ/μ]*100).

Since tomato seeds were grown in different nutritional ME and were germinated in several conditions (GE), the seed germination traits were affected by ME, GE and their interactions (ME × GE). To identify the effect of each component on seed performance traits a two‐way analysis of variance (ANOVA) analysis was performed using Genstat 18 with a significant threshold of 0.05. The contribution of each environmental component (ME, GE and ME × GE) to an individual trait was presented by the sum of squares (SS).

#### 2.3.3 Stability of the genotype rankings over two nutritional maternal environments

For each trait the stability of the genotypes over two nutrient MEs was estimated by calculation of Spearman rank correlation. We used the same approach as performed in previous studies to take the G × E interaction affecting traits into account (Becker & Leon, 1988; Oury et al., 2006).

#### 2.3.4 Principle component analysis

A principal component analysis (PCA) of the RILs and the parents based on the trait measurements was made using the R prcomp function on the correlation between the scaled traits. The first two components of the PCA were plotted using the ggplot2 package (Wickham, 2010).

#### 2.3.5 Correlation analysis

In each ME pairwise Spearman correlation analysis was done between all seed, seedling and seed performance traits using the cor function in R. The values of the correlation and statistically significant level of the correlations was represented as correlation value and false discovery rate (FDR), respectively. Correlation values with FDR ≤ 0.05 were selected to generate a correlation network using Cytoscape v.3.4.0. The NetworkAnalyser tool in Cytoscape was used to obtain further characteristics of the networks.

The correlation between the mean values of each RIL for each trait between two MEs was also calculated using the rcorr R package.

### 2.4 QTL and QTL × E analysis

#### 2.4.1 Linkage analysis

We use the genetic linkage map by Kazmi et al. (2012), in which they used 5,529 SNPs to genotype the RIL population. SNP markers with identical values were removed, leaving 2,251 polymorphic markers. Furthermore, co‐segregating markers were also removed. The remaining 865 unique markers were used for generating the genetic linkage map, which contains 12 individual linkage groups corresponding to the 12 chromosomes of tomato. This map has been constructed using JoinMap 4 (Van Ooijen and Voorrips, 2001) based on recombination frequency and Haldane's mapping function and integrating the existing SNP marker data set for the RILs (Kazmi et al., 2012) (Table S3).

#### 2.4.2 QTL detection

The mean values per RIL of the seed‐, seedling‐ and seed performance‐traits were used for QTL detection. QTL analysis was carried out by genome scan with a single QTL model (scanone) using the r/qtl package (Broman, Wu, Sen, & Churchill, 2003). The Logarithm‐of‐Odds (LOD), physical position, related marker and additive effects of each detected QTL together with phenotypic variation explained by each QTL (explained variance, EV%) were determined. The genome‐wide significant LOD threshold (≥2) was estimated using 10,000 permutation tests (Broman et al., 2003; Doerge & Churchill, 1996). The physical position of the related markers and other characteristics of the QTLs affecting the traits measured for the RIL population grown in the two different MEs are summarized in Table S9. The QTLs for thermo‐tolerance (Th‐T), thermo‐inhibition (Th‐I) and thermo‐dormancy (Th‐D) were previously mapped (Geshnizjani et al., 2018).

#### 2.4.3 QTL × E analysis

The QTL by Environment effect was determined by an ANOVA model in which for each germination trait the model includes; the genetic background (GB), GE, ME and marker under study and their interactions (Phenotype ~ ME * GE * marker + GB). The GB was defined by the RIL identifier. In this way the differences between environments for each individual RIL were taken into account. Phenotype = numerical scored trait (mean value per RIL), ME (LN or HP), GE (Water, NaCl, Mannitol or HT), marker = the *i*th marker from the genetic map (MM or PI) and GB = RIL identifier as the same RILs were measured in the different environments and thus controlling for the RIL background variation. All calculations were done in R and visualised using the R package ggplot2 (Wickham, 2010). Thresholds for QTL by environment effects were determined by permutations (1,000 randomly sampled phenotypic values in the same mapping model). For an additive single maker effect the 0.05 −log10(p) threshold was between 3.6 and 3.9, depending on the trait (3.4–3.5 for 0.1 threshold). For the interaction between the ME and a marker the 0.05 −log10(p) threshold was between 3.3 and 3.6, depending on the trait (3.0–3.3 for 0.1 threshold). For the interaction between the GE and a marker the 0.05 −log10(p) threshold was between 3.2 and 4.2, depending on the trait (3.1–3.3 for 0.1 threshold). For the threeway interaction between the ME, the GE, and a marker the 0.05 −log10(p) threshold was between 3.7 and 3.8, depending on the trait (3.2–3.3 for 0.1 threshold). For convenience the commonly used threshold of −log10(p) > 3 was used, to show significant QTLs in figures.

## 3 RESULTS

To identify the loci involved in variation in tomato seed‐ and seedling‐traits in interaction with different maternal nutritional conditions, HP and LN, we used a population of RILs derived from a cross between a wild (*Solanum pimpinellifolium* [PI]) and a domesticated (*Solanum lycopersicum*, cv. Moneymaker [MM]) tomato species (Voorrips et al., 2000). We mapped QTLs for five seed germination traits under four different GEs, three seed thermo‐dormancy traits (Geshnizjani et al., 2018), two seed morphology traits and four seedling traits (Table 1).

**TABLE 1:** Overview of the traits and the germination environments used in this study

Traits		Germination environments	Codes
Seed germination traits	G_max_	Water	G_max_ water
		NaCl	G_max_ NaCl
		Mannitol	G_max_ Mann
		High temperature	G_max_ HT
	t_10_^−1^	Water	t_10_^−1^ water
		NaCl	t_10_^−1^ NaCl
		Mannitol	t_10_^−1^ Mann
		High temperature	t_10_^−1^ HT
	t_50_^−1^	Water	t_50_^−1^ water
		NaCl	t_50_^−1^ NaCl
		Mannitol	t_50_^−1^ Mann
		High temperature	t_50_^−1^ HT
	AUC	Water	AUC water
		NaCl	AUC NaCl
		Mannitol	AUC Mann
		High temperature	AUC HT
	U_8416_^−1^	Water	U_8416_^−1^ water
		NaCl	U_8416_^−1^ NaCl
		Mannitol	U_8416_^−1^ Mann
		High temperature	U_8416_^−1^ HT
	Thermo‐dormancy	Thermo‐tolerance	Th‐T
		Thermo‐inhibition	Th‐I
		Thermo‐dormancy	Th‐D
Seed and Seedling traits	Seed morphology traits	Seed size	SS
		Seed Wight	SW
	Seedling traits	Fresh weigh of shoot	FWSH
		Dry weigh of shoot	DWSH
		Fresh weigh of root	FWR
		Dry weigh of shoot	DWR

*Note:* t_50_^−1^ and t_10_^−1^, Reciprocal of time to respectively reach 50 and 10% of maximum germination; U_8416_^−1^, Reciprocal of time between 16 and 84% of maximum germination.

Abbreviations: AUC, Area under the germination curve; G_max_, Maximum seed germination percentage.

### 3.1 Variability and heritability of seed and seedling traits

**FIGURE 1:**
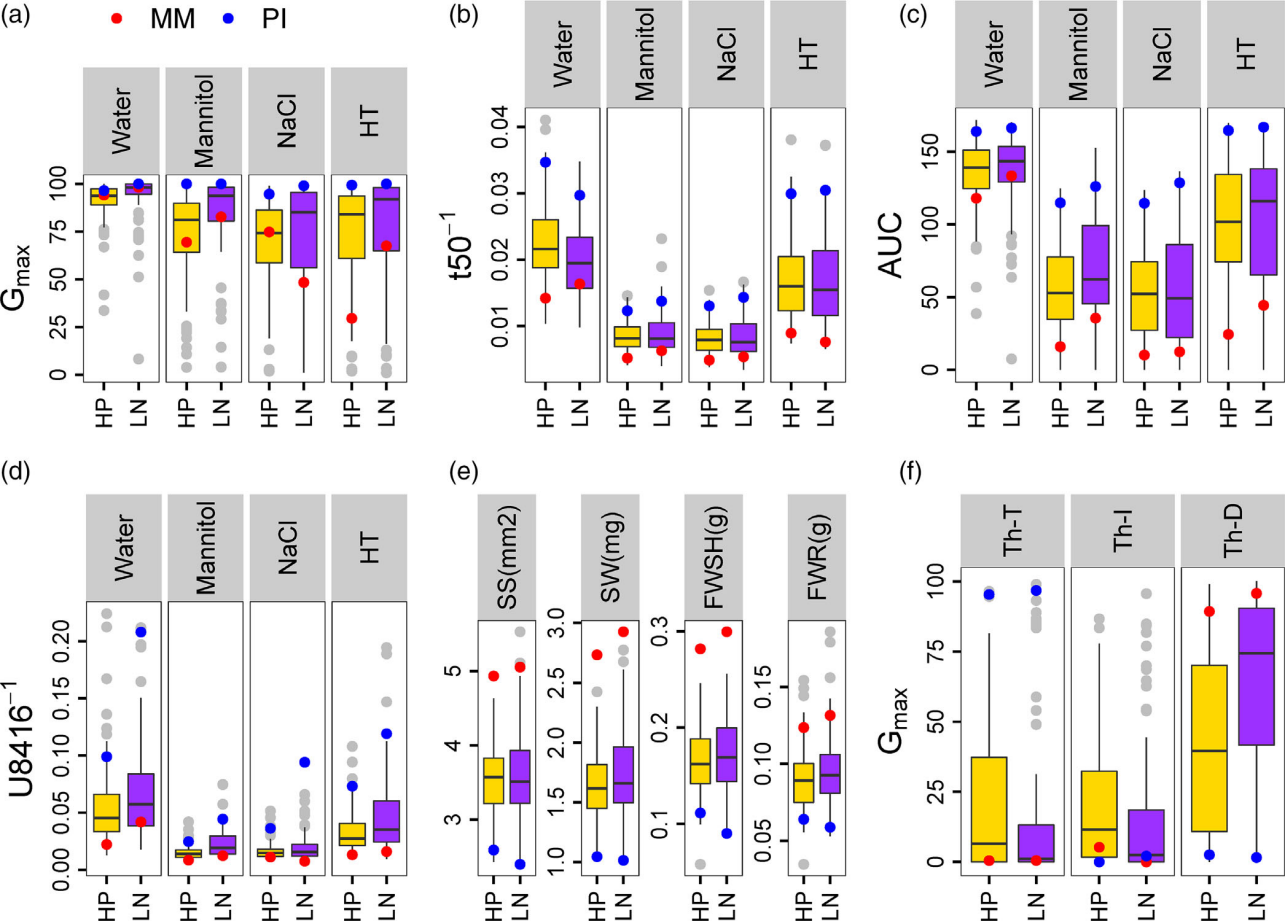
Effect of nutritional maternal environments on seed, seedling and seed germination traits. (a), G_max_, Maximum seed germination percentage; (b), t_50_^−1^, Reciprocal of time to reach 50% of maximum germination; (c), AUC, Area under the germination curve; (d), U_8416_^−1^, Reciprocal of time between 16 and 84% of maximum germination; (e), Seed morphology and seedling traits, SS, Seed size (mm^2^); SW, Seed weight (mg); FWSH, Fresh weight of shoot (g); FWR, Fresh weight of root (g); (f), Response of seed germination to high temperature, Th‐T, Thermo‐tolerance; Th‐I, Thermo‐inhibition; Th‐D, Thermo‐dormancy; HP, High phosphate (in orange); LN, Low nitrate (in purple); Parental lines are shown as colored points: MM, *Solanum lycopersicum* (cv. Moneymaker) in red and (PI), *Solanum pimpinellifolium* in blue; HT, germination condition High temperature; Water, germination condition Water; Mannitol, germination condition Mannitol; NaCl, germination condition Salt. Median of all Recombinant Inbred Lines (RILs) as black line in the boxplot; The hinges correspond to the first and third quartiles (the 25th and 75th percentiles). The whisker extends from the hinge to the largest value no further than 1.5 * IQR from the hinge (where IQR is the inter‐quartile range, or distance between the first and third quartiles). Points indicate outliers beyond the IQR [Colour figure can be viewed at wileyonlinelibrary.com]

**FIGURE 2:**
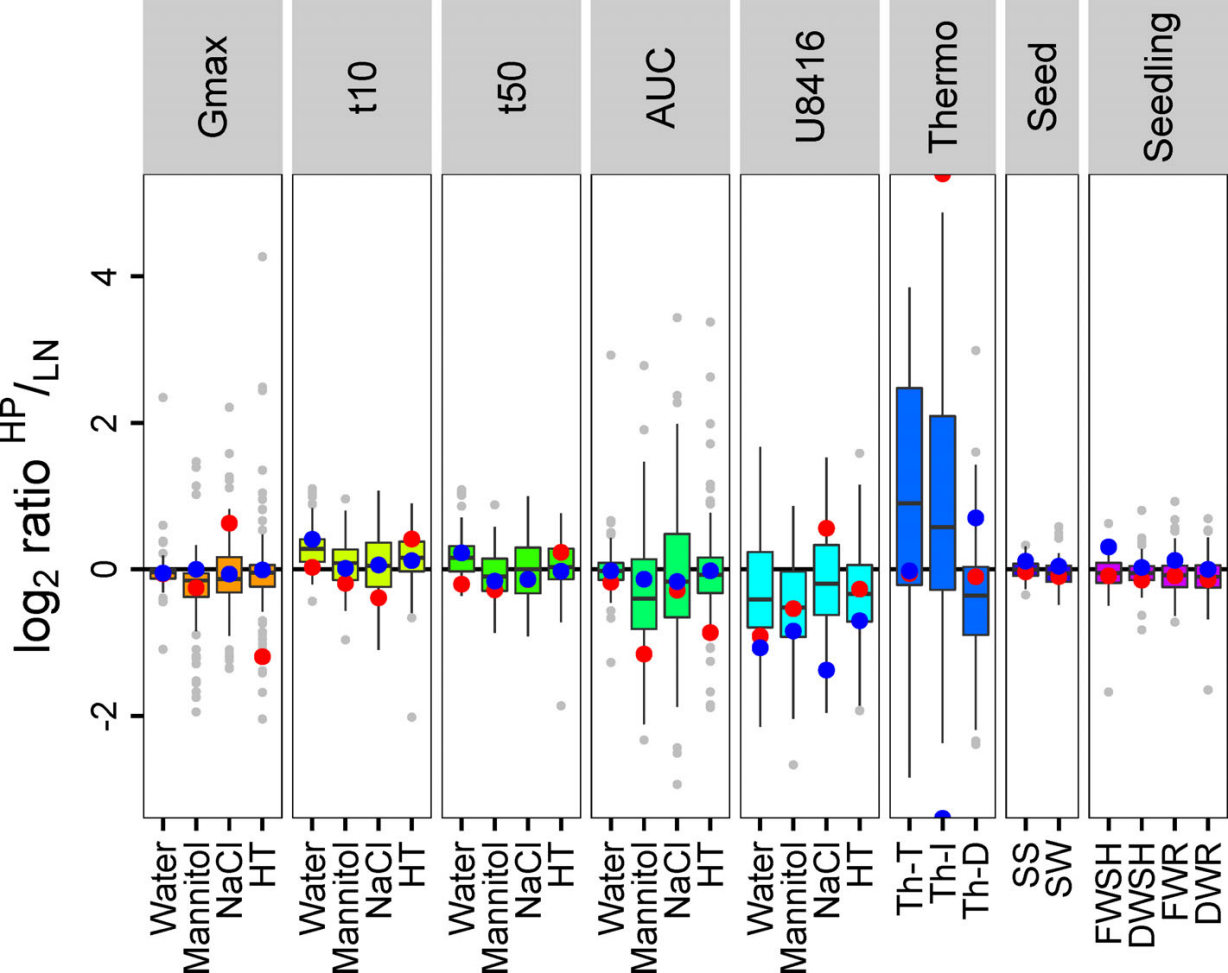
Seed and seed germination trait differences between the maternal environments. Boxplots of the log_2_ ratio of HP:LN per line in each trait. Positive values represent higher phenotypic values under HP and negative values represent higher phenotypic values in LN. HP, High phosphate; LN, Low nitrate; MM, *Solanum lycopersicum* (cv. Moneymaker), also as red points; PI, *Solanum pimpinellifolium*, also as blue points; G_max_, Maximum seed germination percentage; t_50_^−1^, t_10_^−1^, Reciprocal of time to respectively reach 50 and 10% of maximum germination; AUC, Area under the germination curve; U_8416_^−1^, Reciprocal of time between 16 and 84% of maximum germination; Mann, Mannitol; HT, High temperature; Th‐T, Thermo‐tolerance; Th‐I, Thermo‐inhibition; Th‐D, Thermo‐dormancy; SS, Seed size; SW, Seed weight; FWSH, Fresh weight of shoot; DWSH, Dry weight of shoot; FWR, Fresh weight of root; DWR, Dry weight of root. Median of all Recombinant Inbred Lines (RILs) is shown as black line in the boxplot; The hinges correspond to the first and third quartiles (the 25th and 75th percentiles). The whisker extends from the hinge to the largest value no further than 1.5 * IQR from the hinge (where IQR is the inter‐quartile range, or distance between the first and third quartiles). Points indicate outliers beyond the IQR [Colour figure can be viewed at wileyonlinelibrary.com]

In both suboptimal nutritional conditions (HP and LN) most of the traits displayed wide variation for the parental lines MM and PI, as previously observed (Geshnizjani et al., 2019). For the seed germination traits G_max_ and AUC the difference between MM and PI increased under suboptimal germination condition HT, NaCl and Mannitol (Figure 1, Table 2). For most of the traits MM was affected more by suboptimal germination conditions than PI, which confirms the higher susceptibility of MM to stressful conditions, as previously also observed (Geshnizjani et al., 2019) (Figure 1, Figure S1). Calculating the log_2_ ratio of HP:LN showed that in some traits, notably in SS and SW, different maternal nutritional environments hardly affected the parental lines, however in most other traits the phenotypes of the parental lines were differently affected by the HP and LN nutrient environments (Figure 2).

**TABLE 2:** Averages and broad‐sense heritability of seed germination and seedling traits of RILs and their parental accessions *Solanum lycopersicum* (cv. Moneymaker) and *Solanum pimpinellifolium* grown in high phosphate (HP) and low nitrate (LN) conditions

Trait		HP				LN			
		MM	PI	RIL	H^2^ (%)	MM	PI	RIL	H^2^ (%)
G_max_ (%)	Water	94.1	96.5	90.8	77	98.2	100.0	94.4	93
	NaCl	74.8	94.7	69.2	81	48.4	99.0	73.9	85
	Mann.	69.5	100.0	73.2	90	82.7	100.0	85.3	89
	HT	29.5	99.4	73.8	91	67.5	100.0	76.7	89
t_50_^−1^ (×100, h^−1^)	Water	1.42	3.47	2.26	90	1.63	2.97	2.00	91
	NaCl	0.48	1.30	0.82	83	0.53	1.43	0.84	70
	Mann.	0.51	1.23	0.86	87	0.63	1.37	0.90	82
	HT	0.89	2.99	1.71	89	0.76	3.04	1.67	92
t_10_^−1^ (×100, h^−1^)	Water	2.15	4.33	3.08	83	2.11	3.26	2.52	87
	NaCl	0.64	1.65	1.16	83	0.84	1.58	1.15	69
	Mann.	0.76	1.70	1.27	88	0.87	1.68	1.21	84
	HT	1.38	3.92	2.49	88	1.03	3.61	2.23	86
AUC (hrs)	Water	118.0	163.9	135.0	91	133.4	166.3	136.6	93
	NaCl	10.2	114.6	52.0	78	12.4	128.6	55.4	88
	Mann.	15.9	114.8	57.3	85	35.5	126.1	69.7	90
	HT	24.4	164.6	98.6	93	44.4	166.9	100.7	94
U_8416_^−1^ (×100, h^−1^)	Water	2.22	9.89	5.53	66	4.18	20.8	6.76	75
	NaCl	1.11	3.63	1.63	49	0.76	9.41	1.95	54
	Mann.	0.85	2.47	1.51	71	1.24	4.43	2.28	68
	HT	1.32	7.32	3.27	64	1.59	11.9	4.63	69
Dormancy	Th‐T	0.48	95.3	20.5	83	0.50	96.8	13.0	98
	Th‐I	5.23	0.00	23.2	50	0.00	2.09	15.0	94
	Th‐D	89.3	2.54	44.1	86	95.8	1.56	63.1	92
Seed traits	SS	4.93	2.59	3.57	89	5.05	2.40	3.60	94
	SW	0.27	0.10	0.16	89	0.29	0.10	0.17	96
Seedling traits	FWSH	28.2	11.1	16.4	72	30.0	9.00	17.2	78
	DWSH	1.50	0.58	0.92	63	1.66	0.57	0.96	71
	FWR	12.4	6.39	8.94	76	13.2	5.87	9.45	68
	DWR	0.71	0.38	0.53	69	0.79	0.38	0.57	62

Abbreviations: AUC, Area under the germination curve; DWR, Dry weight of root; DWSH, Dry weight of shoot; FWR, Fresh weight of root; FWSH, Fresh weight of shoot; G_max_, Maximum seed germination percentage; HP, High phosphate; HT, High temperature; LN, Low nitrate; Mann, Mannitol; MM, *Solanum lycopersicum* (cv. Moneymaker); PI, *Solanum pimpinellifolium*; RIL, Recombinant Inbred Line; SS, Seed size; SW, Seed weight; Th‐D, Thermo‐dormancy; Th‐I, Thermo‐inhibition; Th‐T, Thermo‐tolerance.

*Note:* H^2^, Broad‐sense heritability (%); t_50_^−1^, t_10_^−1^, Reciprocal of time to respectively reach 50 and 10% of maximum germination; U_8416_^−1^, Reciprocal of time between 16 and 84% of maximum germination.

Moreover, considerable phenotypic variation for some of the traits was found in the RILs for each nutritional environment, this was reflected in the CV ranking from 12 to 120% under HP and 13 to 190% under LN conditions (Figures 1 and 2, Table S4). The largest variation in CV values was perceived in Th‐D followed by AUC and U_8416_ traits indicating high level of variation in these traits. On the other hand, maximum germination percentage (G_max_) of seeds in water showed the lowest percentage of CV which is as expected since most of the RILs germinated almost 100% in water. The log_2_ ratio analysis of HP:LN in RILs exhibited a similar result as the parental line in which several traits like AUC, U_8416_^−1^, Th‐T, Th‐I and Th‐D have been differently affected by HP and LN (Figure 2).

**FIGURE 3:**
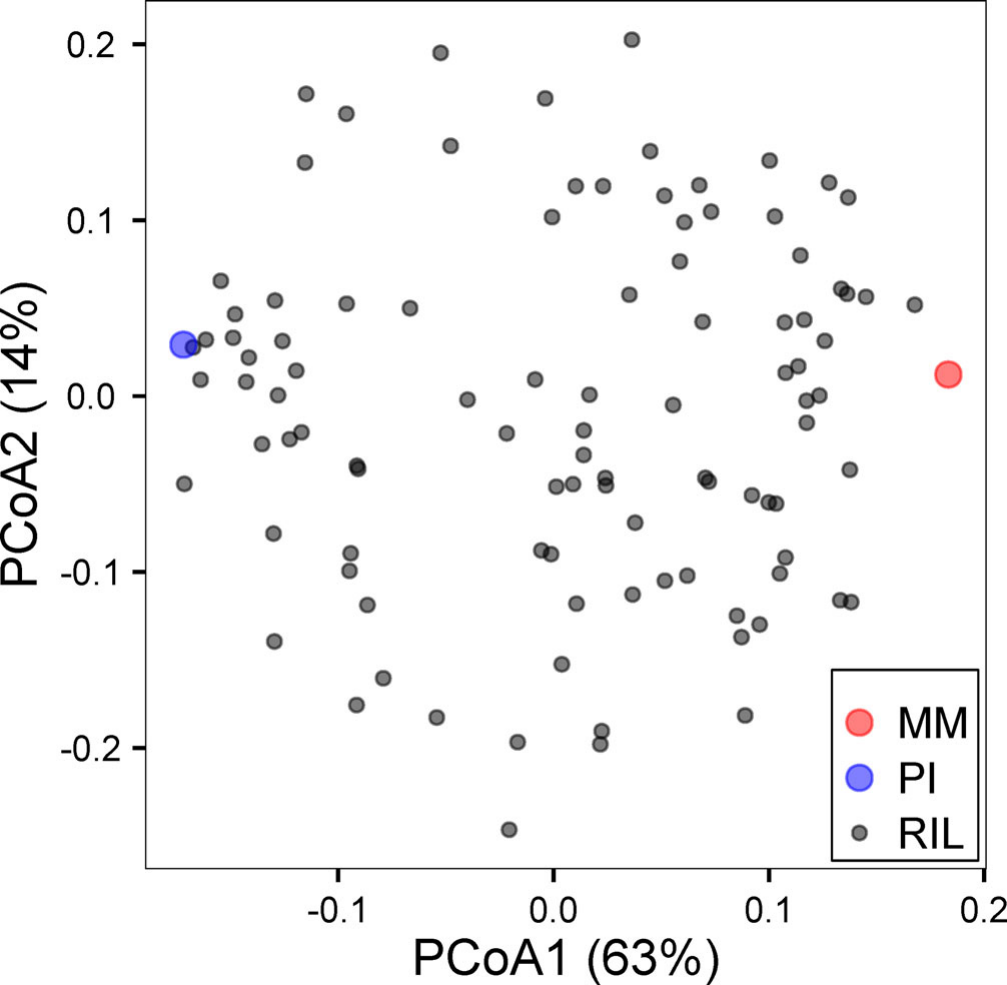
Principle Component Analysis (PCA) of the recombinant inbred and parental lines (*Solanum lycopersicum* (cv. Moneymaker) (MM) in red and Solanum pimpinellifolium (PI) in blue) for all traits in both nutritional maternal environments. Explained variation is shown in the axis titles

The PCA of the RILs and parental lines for all traits in both MEs showed that 63% (PCA1) and 14% (PCA2) of the variation was explained. The PCA plot showed that parental lines in general are flanking the RILs on PCA1 (Figure 3). Similar results have been obtained when considering individual traits where the phenotypes of the RILs are mainly found between the phenotypes of the two parental genotypes; still, the G_max_ under NaCl and the Th‐I traits suggest transgression with some RILs displaying more extremes than their parents. This exemplifies the inheritance from both parental lines to the progenies in which one parent has most positive and the other one has most negative alleles. In a few cases, such as G_max_ water in both nutritional environments, substantial transgression was observed, due to poorly germinating RILs (Figure 1; Table 2).

Broad sense heritability (H^2^) calculated for each trait in both maturation environments was high for most of the traits (with most traits >80% in both environments; ranking from 49 to 91% in HP and 54 to 93% in LN) (Table 2). Taken together this shows that substantial genetic variation exists for these seed traits interacting with the germination as well as the ME.

### 3.2 Genotype ranking and its stability over different nutritional maternal environments

In order to investigate how consistent the phenotypic rankings of the RILs are between the MEs and how large the effect is of the interaction between the genotype and the environment (G × E), the Spearman rank correlation coefficient (Oury et al., 2006) between two suboptimal nutritional MEs was calculated (Table 3, Tables S5 and S6). For phenotypic traits, such as SS and SW, rankings of the genotypes were stable from one ME to another and, thus, Spearman rank correlation values were also high for these traits, which suggests a relatively moderate effect of maternal G × E on seed size and seed weight.

**TABLE 3:** Stability of rankings of the genotypes over the two different nutritional maternal environments

Traits	Spearman rank correlation
Maximum seed germination (G_max_)	0.57
Germination rate (t_50_^−1^)^a^	0.73
Area under the germination curve (AUC)	0.64
Uniformity (U_8416_^−1^)^b^	0.52
Seed size (SS)	0.77
Seed weight (SW)	0.80
Fresh weight of shoot (FWSH)	0.78
Fresh weight of root (FWR)	0.66
Thermo‐dormancy (Th‐D)	0.64

^a^ Reciprocal of time to reach 50% of maximum seed germination.

^b^ Reciprocal of time between 16% and 84% of maximum seed germination.

### 3.3 Germination environments versus maternal environments

By germinating the tomato seeds in optimal (water) and suboptimal conditions, such as salt‐stress (NaCl), osmotic‐stress (Mannitol) and HT stress (35°C), the seed germination traits were affected by their ME, their GE, and their interaction (ME × GE) (Table 4). In comparison to the optimal GE, seed germination traits showed higher variability in suboptimal GE in both MEs (Table 2). For instance, CVs for G_max_ and AUC in water were 12% and 17%, respectively, while they showed significantly higher values in salt‐ (33 and 60% respectively), osmotic‐ (31 and 56% respectively) and HT‐ (35 and 44% respectively) stress (Table 2, Table S4). We observed the same trend for t_10_^−1^ and t_50_^−1^ albeit to a lesser extent. U_8416_^−1^ showed a pattern which was different from other germination traits, where optimal and suboptimal GE show more similar CVs. Taken together, the ME affected seed germination traits less than GE. Although ME did not change the germination traits under optimal GE, it caused a small but significant difference under suboptimal GEs. For example G_max_ exhibited similar CVs under optimal GE in both MEs (HP and LN) whilst under suboptimal conditions they displayed a slight difference in CV (Table 2, Table S4).

**TABLE 4:** Effect of maternal environment (ME), germination environment (GE) and their interaction (ME × GE) on germination traits of tomato seeds

Trait	SS			SL		
	ME	GE	Me×GE	ME	GE	Me×GE
G_max_	0.84	9.82	0.74	**	**	*
t_10_^−1^	0.72	44.29	0.02	*	**	Ns
t_50_^−1^	0.04	50.96	0.14	Ns	**	Ns
AUC	0.00	55.62	0.17	Ns	**	Ns
U_8416_^−1^	2.06	37.31	0.99	**	**	**

*Note:* SS, Sum of square, in each trait represents the proportion of effect of each environmental component (ME, GE and ME×GE) in their total sum of squares; SL, Significant level, represents the significance level of the analysis of variance test for maternal environment, germination environment and the interaction between them; G_max_, Maximum seed germination percentage; t_50_^−1^, t_10_^−1^, Reciprocal time to reach respectively 50 and 10% of maximum germination; AUC, Area under the germination curve; U_8416_^−1^, Reciprocal time between 16 and 84% of maximum germination.

**p value ≤.01; *p value ≤.05; ns, no significant effect.

### 3.4 Trait by trait correlation

**FIGURE 4:**
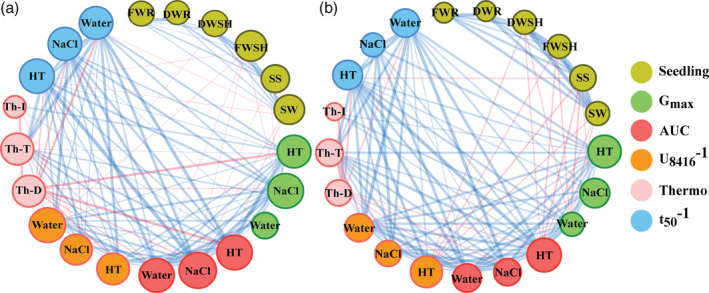
The Spearman correlation coefficient network between the means of phenotypic traits assessed under the two maternal environments: (a), High phosphate; (b), Low nitrate. The false discovery rate cut‐off was 0.05 (FDR ≤ 0.05). The line colour indicates the direction of the correlation, **Red**: Negative correlation, **Blue**: Positive correlation. The width of lines represents the height of the correlation with wider lines indicating higher correlation values. The size of the circles represents the number of edges, bigger circles indicate that a given trait correlates with a higher number of other traits. G_max_, Maximum seed germination; AUC, Area under the germination curve; U_8416_^−1^, Reciprocal time between 16 and 84% of maximum germination; t_50_^−1^, Reciprocal time to reach 50% of maximum seed germination; **Water**, **NaCl** and **HT** are the seed germination environments water, salt and high temperature, respectively; Th‐T, Thermo‐tolerance; Th‐I, Thermo‐inhibition; Th‐D, Thermo‐dormancy; SS, Seed size; SW, Seed weight; FWSH, Fresh weight of shoot; DWSH, Dry weight of shoot; FWR, Fresh weight of root; DWR, Dry weight of root [Colour figure can be viewed at wileyonlinelibrary.com]

To obtain a comprehensive visualization of possible correlations among the phenotypic traits, a correlation network has been generated for each ME (Figure 4). In general, the mean value of all phenotypic traits showed a positive significant correlation between the two suboptimal nutrient environments (HP and LN) (Table S7). Nevertheless, some differences in trait by trait correlation networks between two environments were observed. Some correlations perceived under HP (Figure 4a) were amplified by the LN condition (Figure 4b). For instance, the positive correlations between seed traits (such as, seed size and weight) and seedling quality characteristics (such as, fresh and dry weight of shoot and root) are stronger under the LN condition. In addition, seed and seedling quality traits showed negative association with seed germination traits including G_max_, AUC and U_8416_^−1^, especially in the HT GE, which became visible at the LN condition (Figure 4, Table S8). On the other hand, in both correlation networks, thermo‐dormancy (Th‐D) was negatively correlated with most of the germination traits, including G_max_, AUC and t_50_^−1^ under different GEs (such as water, NaCl and HT). However, they were much more correlated under the high‐phosphate than the low‐nitrate condition (Figure 4, Table S8).

### 3.5 QTL identification for each trait

**FIGURE 5:**
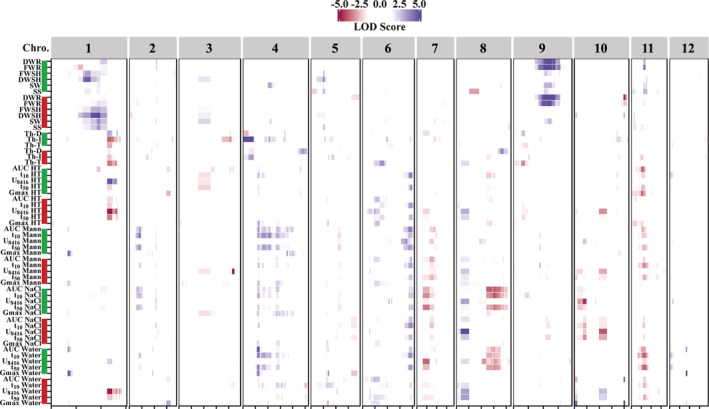
Genomic location of quantitative trait loci (QTLs) detected for seed, seedling and seed performance traits. The **green** and **red** thick lines next to the traits represent the maternal environment: LN and HP, respectively. Chro, Chromosome number; DWR, Dry weight of root; FWR, Fresh weight of root; FWSH, Fresh weight of shoot; DWSH, Dry weight of shoot; SW, Seed weight; SS, Seed size; Th‐D, Thermo‐dormancy; Th‐I, Thermo‐inhibition; Th‐T, Thermo‐tolerance; AUC, Area under the germination curve; t_10_^−1^ and t_50_^−1^, Reciprocal of time to respectively reach 10 and 50% of maximum germination; U_8416_^−1^, Reciprocal of time between 16 and 84% of maximum germination; G_max_, Maximum seed germination percentage; HT, High temperature; Mann, Mannitol. The LOD score scale indicates the significant QTLs. Positive (blue) and negative (red) values represent a larger effect of *Solanum lycopersicum* (cv. Moneymaker) and *Solanum pimpinellifolium* alleles, respectively [Colour figure can be viewed at wileyonlinelibrary.com]

To determine the large effect loci regulating seed, seedling and seed performance traits, QTL analysis of the tomato RIL population was performed. Concerning all traits, with the exception of chromosomes 2, 3, 5 and 12, all chromosomes contain QTLs of which many are co‐located (Figure 5, Table S9). We found 16 QTLs affecting G_max_ under optimal and sub‐optimal GEs of which six were detected in seeds of HP and 10 in LN maternal conditions. For AUC in all GEs, 13 QTLs were found of which nine were co‐locating with the ones affecting G_max_ on chromosomes 1, 4, 5, 10 and 11. With the exception of two QTLs on chromosome 6 and 10 discovered for the HP environment, all other QTLs regulating AUC were associated with the LN maternal condition. The result showed that t_10_^−1^ and t_50_^−1^ in all GEs and both MEs are regulated by almost the same QTLs which is not surprising as they are highly correlated traits. In total 18 QTLs were detected for t_10_^−1^ and t_50_^−1^ on chromosomes 2, 4, 6, 7, 8 and 11 which are also largely related to the LN ME (Figure 5, Table S9).

For SS and SW, three and four QTLs were found respectively. The co‐locating QTLs for these two seed traits for the HP ME were detected on chromosome 1. A co‐located QTL was also found for seedling quality in the same ME. Furthermore, another QTL related to seedling quality on chromosome 9 is co‐locating with seed traits such as SW.

There is a strong QTL on chromosome 1 regulating thermo‐dormancy traits in both MEs. This QTL affects both Th‐T and Th‐I traits in the same direction, while antagonistically regulating Th‐D (Figure 5, Table S9). This QTL is co‐locating with seed germination traits, such as t_50_^−1^ and U_8416_^−1^ under HT germination conditions.

For seed germination traits under salt and mannitol germination conditions a co‐located QTL is found on chromosome 7. This might be related to the fact that both salt and mannitol cause osmotic stress for seeds and thus seed germination could be regulated by similar mechanisms. On the other hand, we have also identified QTLs on chromosome 8 which are present in the LN ME only. Also, on chromosome 11, a QTL was detected for seed germination in both maternal environmental conditions, which was stronger when maternal plants were cultivated in LN conditions. These QTLs might have been detected as a consequence of genotype by environment interactions (G × E).

### 3.6 QTL by environment

**FIGURE 6:**
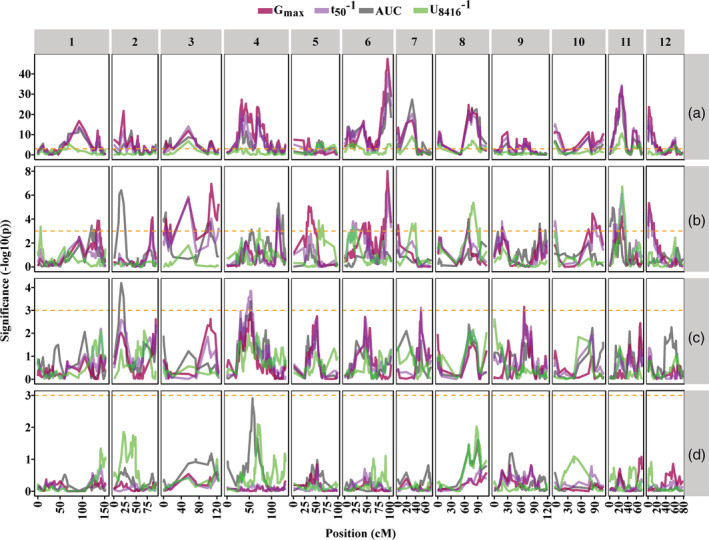
Profiles of the QTLs regulating the seed germination traits. (a), QTLs detected in all maternal and germination environments; (b), QTLs with significant effect of germination environment (GE); (c), QTLs with significant effect of maternal environment (ME); (d), QTLs with significant effect of GE × ME; G_max_, Maximum seed germination percentage (in red); t_50_^−1^, Reciprocal of time to reach 50% of maximum germination (in purple); AUC, Area under the germination curve (in gray); U_8416_^−1^, Reciprocal of time between 16 and 84% of maximum germination (in green). QTL, quantitative trait loci [Colour figure can be viewed at wileyonlinelibrary.com]

**FIGURE 7:**
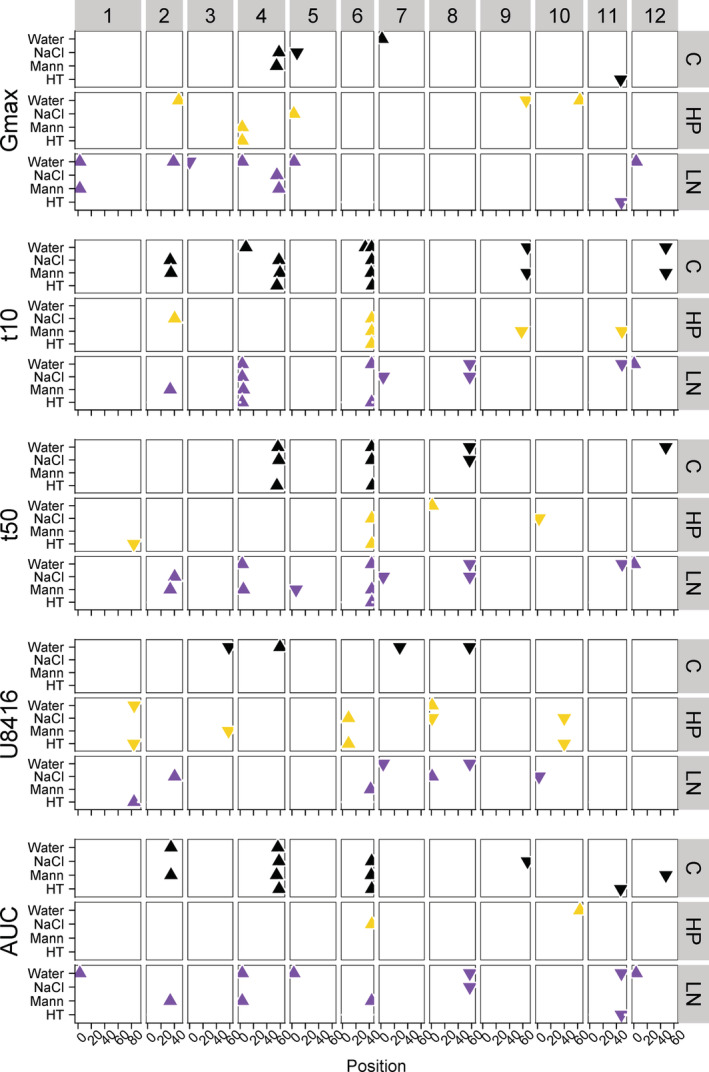
Comparison between the QTLs found in the sub‐optimal maternal conditions in this study and the QTLs found in the control maternal conditions from Kazmi et al., 2012. Chromosomes are indicated on top. Maternal conditions are shown on the right and indicated by colors (control conditions in black, HP in yellow and LN in purple), phenotypes are shown on the left. Germination environments are shown on the y‐axis and the position on the genome on the x‐axis (in Mbp). Triangle pointed upwards means the MM allele increased the phenotype compared to the Pimp allele and vice versa for the triangle pointed downwards. QTL, quantitative trait loci [Colour figure can be viewed at wileyonlinelibrary.com]

Generally, when different environments are studied simultaneously, detected QTLs can be affected by several environments. The QTL by Environment interaction (QTL × E) can describe such effects. In this study seeds were grown under two MEs, HP and LN and germinated in optimal (water) and three suboptimal conditions: osmotic (NaCl and mannitol) and HT stress. Therefore, in each seed germination trait the environmental component of QTL × E can be explained by either the ME or the GE and their interaction (ME × GE). We identified the QTLs affected by the environments and also decomposed the environmental effect into the different environmental components; GE, ME and their interaction (Figure 6). Figure 6a shows the QTLs regulating the seed germination traits independently from the environments. Those QTLs were detected through all the maternal and GEs. With the exception of chromosomes 5, 9 and 10, the rest of the chromosomes displayed several QTLs strongly regulating seed germination traits including G_max_, t_50_^−1^, AUC and U_8416_^−1^. As an example, the QTL at the bottom of chromosome 6 significantly affected G_max_, t_50_^−1^ and AUC regardless of the different environments under which seeds had developed or were germinated (Figures 6 and 7, Figure S2). On the other hand, some of the QTLs regulating seed germination traits are significantly influenced by the environment. For example the QTL located near the top of chromosome 2, which regulates AUC, was significantly affected by GE and to a lesser extent by ME (Figures 6 and 7, Figure S2). We have observed that GE showed generally more effects on QTLs than the ME. This result is in accordance with the observed variance between ME and GE in which seed germination traits showed higher variance in different GEs in comparison with different MEs. GE affects QTLs related to t_10_^−1^ and t_50_^−1^, located on chromosomes 3, 6 and 11. Some QTLs affecting U_8416_^−1^ on chromosomes 8 and 11 were also affected by the GE (Figure 6, Figure S2). In comparison with GE, ME showed a less pronounced effect on the QTLs. Although the detected QTLs were sometimes affected by either maternal or GEs, we only found a suggestive interaction of a QTL, GE and ME (Figure 6, Figure S3). Comparing the QTLs found in the stressfull MEs, HP and LN, to QTLs found in control conditions from Kazmi et al. (2012) (Figure 7) shows that the majority of QLTs is ME specific. The QTLs are often shared between GE yet many QTLs occur only in specific combinations of maternal and GE.

## 4 DISCUSSION

In this study we have used the genetic variation in a tomato RIL population to study how the genotype, ME and GE, including their interactions affects seed‐ and seedling‐ quality traits. A tomato RIL population was grown in two different MEs with suboptimal nutritional conditions, low nitrogen and HP. The seed produced in these environments were used to study the effect of genetic variation and variation in the ME on seed quality and seedling establishment related traits. Nitrogen and phosphorus are two key elements required for plant growth (Schachtman, Reid, & Ayling, 1998; Urbanczyk‐Wochniak & Fernie, 2004). Hence, their non‐optimal concentrations in mother plants may seriously affect the produced seed and the seedlings from those seeds. Moreover, the effect of the GE on the seedling establishment was further studied by observing these traits in four different GEs.

Although several studies have been conducted previously on the effect of abiotic stresses, such as drought and salt stress on seed quality (Asins, Raga, Roca, Belver, & Carbonell, 2015; Foolad, 2004; Foolad, Zhang, & Subbiah, 2003), studies of the effect of maternal nutritional conditions on the produced seed and seedling traits are scarce (Geshnizjani et al., 2019; He et al., 2014). By exploiting the natural variation observed in a tomato RIL population obtained from a cross between *Solanum lycopersicum* (cv. Moneymaker) and *Solanum pimpinellifolium*, we identified several loci controlling seed and seedling traits related to suboptimal nutritional seed maturation conditions, as well as suboptimal germination conditions.

### 4.1 How are seed and seedling traits correlated?

Breeders and producers often are interested in seed traits such as t_50_^−1^ and seedling traits such as ability to produce normal and healthy seedlings. Furthermore, traits such as germination percentage and uniformity of germination, may also pose an important focus for breeders. The AUC (combining germination rate [t_50_] and percentage [G_max_]) will determine how fast seeds will germinate to a certain level, which directly affects further establishment of seedlings. On the other hand, seedling properties such as shoot and root weight determine how fast seedlings can penetrate the soil and start nutrient uptake and how fast the above ground tissues develop to provide required assimilates through photosynthesis. All together these factors determine seed and seedling vigour. Correlation of seed traits (SS and SW) with seed performance (rate of seed germination and uniformity) and with seedling traits have been studied before. Many studies have implied a direct relation between SS and SW and better seedling growth (Doganlar, Frary, & Tanksley, 2000; Khan et al., 2012; Nieuwhof, Garretsen, & Oeveren, 1989). This can be due to the amounts of reserve food which are deposited in seeds during seed development and maturation. Bigger tomato seeds produce seedlings with higher weight (Geshnizjani et al., 2019; Khan et al., 2012; Nieuwhof et al., 1989). Our results confirm the relation of SS and SW with seedling quality and establishment. In both suboptimal nutritional maternal conditions SS and SW were significantly influencing seedling quality traits. However, this correlation was most obvious in the LN nutritional condition. Such a strong correlation between seed and seedling traits suggests a similar genetic architecture, whereas the environment can partially affect such relations. In the former study in which the same RIL population was grown in standard conditions, similar correlations have been found between seed and seedling size. However, there was no obvious correlation between SS and seed germination traits (Khan et al., 2012). This contradicts our findings in which significant negative correlations were found between SS and seed performance traits such as G_max_, t_50_^−1^, AUC and U_8416_^−1^ in both nutritional conditions. Such a negative correlation was even more apparent if seeds were germinated at HT. Such a discrepancy may be caused by the MEs under which seeds developed and matured. Khan et al. (2012) grew the RILs under optimal environment while suboptimal maturation environments were used in this study. Hence it is postulated that the stressful environments that we used affect the correlation of the seed size and seed germination traits such as G_max_ and t_50_^−1^.

The negative correlation that we found between SS and seed performance has been reported previously in tomato. The inheritance of germination time factors (e.g. t_50_^−1^) was negatively correlated with SS, implying that smaller seeds take longer to germinate (Whittington, 1973). We also have found collocated QTLs for SS and seed performance traits such as G_max_ and t_50_^−1^ on chromosome 11 which antagonistically affected the traits under study. Such co‐locating QTLs might be an indication for the same regulatory mechanism for these traits.

### 4.2 Breeding of crops

In general, a breeding strategy is highly dependent on genotype by environment interactions and the heritability level. Detection of a high correlation between the performance of genotypes in the different MEs may simplify the breeding strategy as it is then not required to select different genotypes for implementation into a breeding program. It has been mentioned previously that genotype re‐ranking per trait in different environments is an indication of genotype by environment interaction (G × E) (Oury et al., 2006). Considering this, good breeding traits are the ones with the lower G × E effects. The results of the Spearman correlation analysis show that genotype re‐ranking for most of the studied traits did not occur, therefore traits were limited affected by G × E (Table 5). According to the results we would expect a successful breeding process of the traits such as SS, SW, t_50_^−1^ as well as seedling traits such as FWSH due to their high correlation value. In contrast, breeding for traits like U_8416_^−1^ with a low correlation value would encounter difficulties because of the feasible influence of the G × E interaction. Furthermore, the genotype ranking per trait demonstrated that from the first 10 genotypes per trait some are consistent between two MEs, which is dependent on the trait. Regarding the seed performance traits, with the exception of G_max_, within the rest of the traits including t_50_^−1^, AUC and U_8416_^−1^ three genotypes (207, 250 and 289) showed a consistent high ranking level between MEs (Table 5). Hence these high‐ranking genotypes may be selected for breeding programs for seed performance. Through the seed traits such as SS and SW we also found three stable genotypes (235, 238 and 258) between two MEs which could be considered as good candidates for further breeding of seed traits (Table 5).

**TABLE 5:** The 10 genotypes with the highest value per trait within two nutritional maternal environments

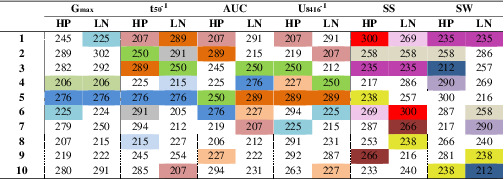

Abbreviations: AUC, area under the germination curve; G_max_, maximum seed germination; HP, High Phosphate; LN, Low Nitrate; SS, Seed size; SW, Seed weight.

*Note:* t_50_^−1^, Reciprocal of time to reach 50% of maximum seed germination; U_8416_^−1^, Reciprocal of time between 16% and 84% of maximum germination. The consistent genotypes are highlighted.

### 4.3 QTL and QTL × E detection

In general, QTL detection depends on several factors such as trait heritability, population type, number of lines and genetic map quality (Mackay, 2001; Mackay, Stone, & Ayroles, 2009). Controlled growth conditions of the plants together with controlled conditions of performed experiments resulted in identification of traits with high heritability values in our study. Substantial variation found between the parental lines and the 100 RILs provided us with a powerful tool for analysing the genetical background of traits by QTL analysis. QTL analysis ultimately resulted in identification of several interesting QTLs, regulating seed and seed performance traits, as well as seedling characteristics. In this, we have discovered more QTLs with high explained variance at LN ME as compared to HP (Figures 5 and 7). Such a result could indicate that more physiological mechanisms and, subsequently, more genes are involved in plant adaptation to a LN environment. Many of the identified QTLs in this study have been reported previously for the same population, but under standard conditions only (Kazmi et al., 2012; Khan et al., 2012) (Figure 7). For example the QTL that we have found at the end of chromosome 6, predominantly regulating the t_50_^−1^ trait in both MEs, was also detected in the standard condition. In addition, we have identified more environment‐specific QTLs which were detected exclusively in one of the environments. These QTLs are more interesting from scientific point of view, however, QTLs detected in all different environments which may be considered as robust QTLs are the most interesting ones for further analysis for breeders and producers. These stable QTLs could regulate the traits independent from the growth environment. Further analysis, such as fine mapping, would ultimately result in identification of gene(s) regulating the analysed traits. As an example, many studies carried out so far to identify the genetic loci regulating SW in tomato have resulted in the identification of several QTLs (Doganlar et al., 2000; Grandillo & Tanksley, 1996; Khan et al., 2012; Tanksley, Medina‐Filho, & Rick, 1982; Weller, Soller, & Brody, 1988). An interesting QTL which is common in different reports, and for which the causal gene has been identified, is present on chromosome 4 (Khan et al., 2012; Orsi & Tanksley, 2009). A co‐locating QTL also appeared in our population grown under LN nutritional condition. Under HP nutritional condition the QTL was just below threshold (Figure 5).

Studies of the interactions of QTL by environment have been carried out previously in different crops including tomato and rice taking a relatively simple strategy (Lu et al., 1997; Paterson et al., 1991). Plants were grown in different environments, QTL analysis was performed for individual environments and finally the results obtained from the different environments were compared with each other. In this study we also report the interactions between the QTLs, the nutritional environment, and the GE. We used a more complex strategy which has been applied previously for other species and/or environments (Des Marais et al., 2013; Snoek et al., 2015; van Eeuwijk, Bink, Chenu, & Chapman, 2010). In this method QTLs are directly studied in several environments. Although there is considerable overlap between the simple and more complex strategies, the second method enhances the statistical analysis resulting in higher LOD values and higher chances of finding significant QTLs (Tétard‐Jones, Kertesz, & Preziosi, 2011). According to our results (Figure 6) we have detected some QTLs with significant QTL × E. The interaction between QTLs and environment are mostly applied by GEs, which indicates that most QTLs are regulating the tomato seed germination traits independently from the MEs. Therefore, we conclude that in comparison with the nutritional ME, the GE must be considered as the more important factor for seed performance in tomato. Nevertheless, also some QTLs show interaction with the ME and even some suggestive QTLs in which the interaction between the ME and GE could play a role.

Taken together, our results provide the genetic architecture of the effects of the ME on seed and seedling traits. These results could be further implemented in tomato breeding programs. We also suggest fine mapping of detected QTLs to narrow down the quantitative genetic loci and ultimately identify the causal gene(s). These can be the start to investigate more in‐depth details of the molecular regulation of seed germination performance under different maternal and GEs.

## CONFLICT OF INTEREST

The authors declare no conflicts of interest.

## AUTHOR CONTRIBUTIONS

Henk W. M. Hilhorst and Wilco Ligterink conceived the study, Nafiseh Geshnizjani, Leo A. J. Willems, Juriaan A. Rienstra performed the experiments, Basten L. Snoek, Harm Nijveen and Nafiseh Geshnizjani analysed the data. Nafiseh Geshnizjani, Basten L. Snoek and Wilco Ligterink wrote the paper with help from all co‐authors.

## Supporting information


**FIGURE S1** Effect of nutritional maternal environments on seed and seedling traits. **t**_**10**_^**−1**^, Reciprocal of time to reach 10% of maximum germination; **DWSH**, Dry weight of shoot; **DWR**, Dry weight of root.
**FIGURE S2.** Heatmap of QTLs regulating the seed germination traits. (a) QTLs detected in all maternal and germination environments; (b) QTLs with significant effect of germination environment (**GE**); (c) QTLs with significant effect of maternal environment (**ME**); (d) QTLs with significant effect of **GE×ME; G**_**max**_, Maximum seed germination percentage; **t**_**50**_^**−1**^ **and t**_**10**_^**−1**^, Reciprocal of time to reach 50 and 10% of maximum germination, respectively; **AUC**, Area under the germination curve; **U**_**8416**_^**−1**^, Reciprocal of time between 16 and 84% of maximum germination.
**FIGURE S3**: Allelic effects of QTL at chromosome 4 at ~6.2 M basepairs, on AUC per maternal and germination environment. MM in red and PI in blue.

**TABLE S1** Nutrient conditions of mother plants after flowering.
**TABLE S2.** Average trait values per RIL per phenotype.
**TABLE S3.** Marker data per RIL per SNP.
**TABLE S4.** Value of Coefficient Variation (CV%) per each trait across the tomato Recombinant Inbred Line (RIL) population containing 100 lines.
**TABLE S5.** The ranking of the genotypes for seed germination traits within each maternal environments.
**TABLE S6.** The ranking of the genotypes for seed and seedling traits within each maternal environments.
**TABLE S7.** Correlation and *P* value of the traits between two nutritional maternal environments: high phosphate and low nitrate.
**TABLE S8.** Significant correlation values between the seed and seedling quality traits in each maternal environment. Low nitrate (LN) and high phosphate (HP).
**TABLE S9.** The characteristics of detected QTLs. The related marker, physical position and the effect of the detected QTLs related to seed, seedling and seed performance traits in tomato RIL population.
**TABLE S10.** The authors declare no conflicts of interest. List of the nutrient solutions with their concentrations used for different growing environments of tomato plants.
