# Supplementary material for: Detection of QTLs for genotype × environment interactions in tomato seeds and seedlings
Source: Plant Cell Environ. 2020 Jun 11;43(8):1973–88. doi: 10.1111/pce.13788 (PMC7496158; doi:10.1111/pce.13788)
Supplement: Supplementary file 1 — FIGURE S1 Effect of nutritional maternal environments on seed and seedling traits. t 10 −1, Reciprocal of time to reach 10% of maximum germination; DWSH, Dry weight of shoot; DWR, Dry weight of root. FIGURE S2. Heatmap of QTLs regulating the seed germination traits. (a) QTLs detected in all maternal and germination environments; (b) QTLs with significant effect of germination environment (GE); (c) QTLs with significant effect of maternal environment (ME); (d) QTLs with significant effect of GE×ME; G max, Maximum seed germination percentage; t 50 −1 and t 10 −1, Reciprocal of time to reach 50 and 10% of maximum germination, respectively; AUC, Area under the germination curve; U 8416 −1, Reciprocal of time between 16 and 84% of maximum germination. FIGURE S3: Allelic effects of QTL at chromosome 4 at ~6.2 M basepairs, on AUC per maternal and germination environment. MM in red and PI in blue. [file PCE-43-1973-s001.docx]

| 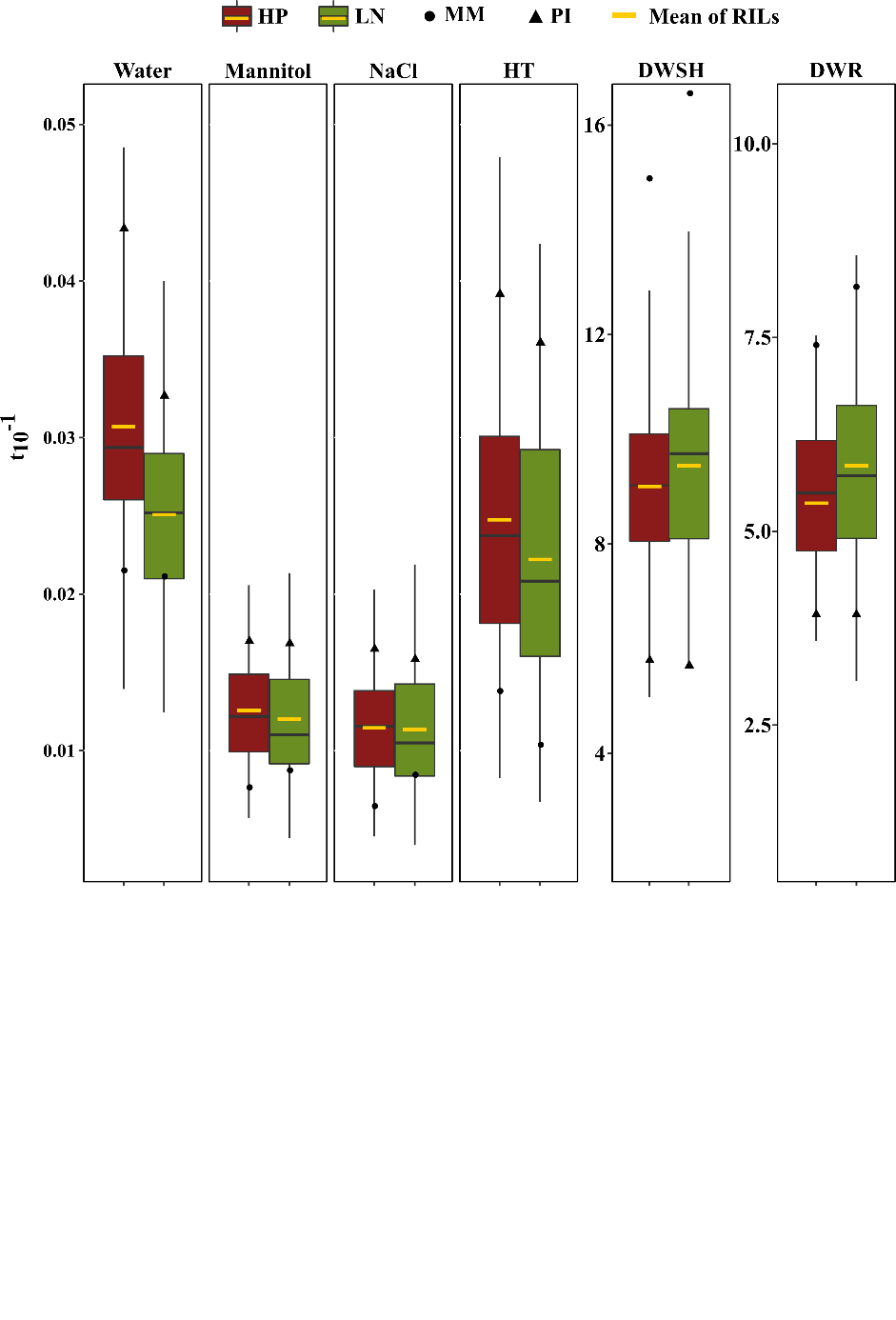 |
| --- |
| **Supplemental Figure 1.** Effect of nutritional maternal environments on seed and seedling traits. **t_10_^-1^,** Reciprocal of time to reach 10% of maximum germination; **DWSH,** Dry weight of shoot; **DWR,** Dry weight of root. |

| 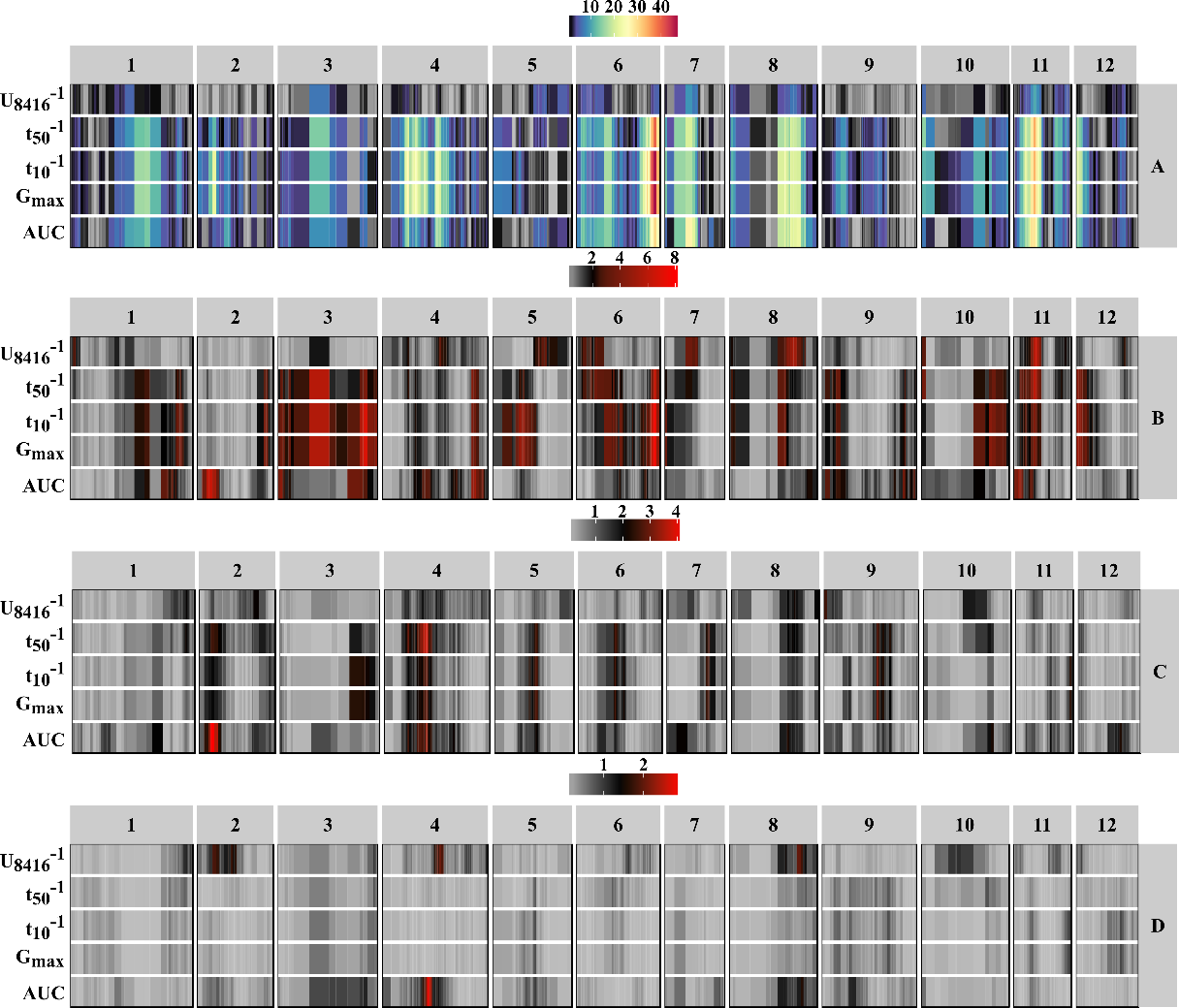 |
| --- |
| **Supplemental Figure 2.** Heatmap of QTLs regulating the seed germination traits. **A,** QTLs detected in all maternal and germination environments; **B,** QTLs with significant effect of germination environment **(GE)**; **C,** QTLs with significant effect of maternal environment **(ME)**; **D,** QTLs with significant effect of **GE×ME;** **G_max_**, Maximum seed germination percentage; **t_50_^-1^ and t_10_^-1^**, Reciprocal of time to reach 50 and 10% of maximum germination, respectively; **AUC**, Area under the germination curve; **U_8416_^-1^**, Reciprocal of time between 16 and 84% of maximum germination. |

**Supplemental Figure 3**: Allelic effects of QTL at chromosome 4 at ~6.2M basepairs, on AUC per maternal and germination environment. MM in red and PI in blue.
